# Validation of Monte Carlo dose calculation algorithm for CyberKnife multileaf collimator

**DOI:** 10.1002/acm2.13481

**Published:** 2021-12-01

**Authors:** Maude Gondré, Fanny Marsolat, Jean Bourhis, François Bochud, Raphaël Moeckli

**Affiliations:** ^1^ Institute of Radiation Physics Lausanne University Hospital and Lausanne University Lausanne Switzerland; ^2^ Radio‐Oncology Department Lausanne University Hospital and Lausanne University Lausanne Switzerland

**Keywords:** commissioning, CyberKnife, Monte Carlo dose algorithm, multileaf collimator

## Abstract

**Purpose:**

To commission and evaluate the Monte Carlo (MC) dose calculation algorithm for the CyberKnife equipped with a multileaf collimator (MLC).

**Methods:**

We created a MC model for the MLC using an integrated module of the CyberKnife treatment planning software (TPS). Two parameters could be optimized: the maximum energy and the source full width at half‐maximum (FWHM). The optimization was performed by minimizing the differences between the measured and the MC calculated tissue phantom ratios and profiles. MLC plans were calculated in the TPS with the MC algorithm and irradiated on different phantoms. The dose was measured using an A1SL ionization chamber and EBT3 Gafchromic films, and then compared to the TPS dose to obtain dose differences (Δ*D*). Finally, patient‐specific quality assurances (QA) were performed with global gamma index criteria of 3%/1 mm.

**Results:**

The maximum energy and source FWHM showing the best agreement with measurements were 6.4 MeV and 1.8 mm. The output factors calculated with these parameters gave an agreement within ±1% with measurements. The Δ*D* showed that MC model systematically underestimated the dose with an average of −1.5% over all configurations tested. For depths deeper than 12 cm, the Δ*D* increased, up to −3.0% (maximum at 15.5 cm depth).

**Conclusions:**

The MC model for MLC of CyberKnife is clinically acceptable but underestimates the delivered dose by an average of −1.5%. Therefore, we recommend using the MC algorithm with the MLC only in heterogeneous regions and for shallow‐seated tumors.

## 1 INTRODUCTION

The CyberKnife (CK) is designed to deliver stereotactic radiosurgery (SRS) treatments, as well as stereotactic body radiation therapy (SBRT) using a robotic arm and real‐time adaptive delivery. Three types of collimators are available. The fixed collimators provide fixed diameters of 7.5–60 mm, the Iris collimator provides variable circular apertures of 7.5–60 mm, and the multileaf collimator (MLC) is for treating larger irregularly shaped lesions with a maximum treatment size of 115.0 × 100.1 mm^2^. Three dose calculation algorithms are available in the Precision TPS of CK. The RayTracing algorithm is available for the fixed and Iris collimators and uses effective path length to account for density variations. This algorithm ignores scatter changes due to local heterogeneities and thus, the calculated dose may result in inaccuracies near density interfaces.^1^ This means that it overestimates the calculated dose in low‐density tissue, such as the lungs, making the RayTracing algorithm clinically unsuitable for lung treatments. The Finite Size Pencil Beam (FSPB) algorithm is available for the MLC only. The beam is divided into small rectangular pencil beams and the dose distribution of each beam is calculated by the convolution of energy fluence and dose deposition kernel. The sum of all pencil beams contributions gives the dose at a given point.^2^, ^3^ Because it uses effective path length to calculate doses, the FPSB algorithm is also not suited in case of heterogeneity, like the RayTracing algorithm. The Monte Carlo (MC) algorithm was first available for the fixed and Iris collimators and is now for the MLC. The MC algorithm accounts for lateral electronic scatter and lateral electronic disequilibrium. This makes the dose calculations more accurate, especially in a heterogeneous medium.^4^ Its accuracy has been evaluated for the fixed collimator and the difference between measured and MC calculated doses was within ±3%.^5^ The objective of this work was to commission and validate the MC calculation algorithm for the MLC with a special emphasis on lung treatments.

## 2 MATERIALS AND METHODS

### 2.1 Beam modeling for MC algorithm

We used the Precision TPS version 2.0.1.1 to create the beam model for RayTracing (fixed and Iris collimators), FSPB (MLC), and MC (all collimators) algorithms. To create the MC model, we measured tissue phantom ratios (TPRs), profiles, and output factors for 11 MLC square field sizes ranging from 7.6 × 7.7 to 115.0 × 100.1 mm^2^. The acquisition of these data (referred hereafter as “measurements”) was performed with a PTW 60018 stereotactic diode (PTW, Germany) in a water tank. Commissioning measurements of TPRs, profiles in the *X* and *Y*‐directions, and output factors were acquired in 2015 in order to implement FSPB algorithm for the MLC. We were able to use the same measurements for implementing the MC model. However, at the time of these initial measurements, Accuray recommended the use of stereotactic diode, despite its inaccurate dose measurements in tails of profiles due to its nonwater equivalence. This constitutes a limitation of our study that will be discussed further below. The model was iteratively built by reducing the differences between the measured and the MC calculated TPRs and profiles. The maximum energy (*E*_max_), which represents the maximum value of the energy spectra, and the source size (*S*) defined by its full width at half‐maximum (FWHM) were the two parameters that could be optimized. The TPRs were used to optimize *E*_max_ and the profiles were used to optimize *S*. In fact, although *S* has a minor effect on the TPRs, *E*_max_ has a consequent impact on profiles^6^ and therefore TPRs for all field sizes and depths should be calculated with the chosen *E*_max_ in order to calculate profiles. This is a limitation because if evaluating the influence of different *E*_max_ on profiles is needed, all TPRs should be calculated several times with different *E*_max_. The operation would need to be repeated for different *S*. This process would have been very time consuming and would not have been compatible with clinical time constraints, and this forced us to optimize *E*_max_ and *S* independently.

#### 2.1.1 Determination of the optimized *E*_max_

Energy spectra that represent the distribution of initial photon energies and that are characterized by *E*_max_ are pre‐calculated in the TPS. They serve as an input to calculate the TPRs. Accuray recommended starting the optimization by calculating the TPRs for small (15.4 × 15.4 mm^2^) and large (84.6 × 84.7 mm^2^) field sizes, and for five depths (10, 15, 100, 200, and 300 mm).^3^ The MC calculation statistical uncertainty was set to 0.5% and S to 1.8 mm, as recommended by Accuray.^3^ We performed the optimization in two successive phases. *First phase*: TPRs were calculated with *E*_max_ ranging from 6.3 to 6.8 MeV with intervals of 0.1 MeV for field sizes and depths defined above. Measured and MC calculated TPRs were compared using the metrics defined hereafter. *Second phase*: from the differences obtained between MC calculated and measured TPRs in the first phase, two *E*_max_ (6.4 and 6.5 MeV) were considered as potentially optimal. These two *E*_max_ were therefore used to calculate TPRs for all field sizes and depths available in the Precision TPS. Additionally, TPR differences for a reduced set of available field sizes and depths, considered as more clinically relevant, were also computed (field sizes from 7.6 × 7.7 mm^2^ to 38.4 × 38.5 mm^2^ and depths of 10, 15, 20, 50, 100, and 200 mm). For this second phase, we used the same metrics as for the first phase in order to define the optimal *E*_max_ between 6.4 and 6.5 MeV. The metrics used to guide the discrimination of the optimal *E*_max_ were (1) the mean difference between measured and MC calculated TPRs, and (2) the percentage of calculated points of the TPRs with a difference from measurements below ±1% (ΔTPR%). These two metrics were used as follows: if an *E*_max_ gave the lowest mean difference as well as the highest ΔTPR% among all tested *E*_max_, it was considered optimal. We preferred a higher ΔTPR% so that if the mean difference obtained with an *E*_max,1_ was lower than with an *E*_max,2_, but the ΔTPR% was higher with *E*_max,2_, then *E*_max,2_ was preferred to *E*_max,1_. We favored a higher ΔTPR% because both the Swiss Society of Radiology and Medical Physics (SSRMP) recommendation for quality control of medical accelerators^7^ and the American Association of Physicists in Medicine (AAPM) guidelines on linear accelerator performance tests^8^ require a tolerance of 1% on TPRs dose differences (DDs).

Additionally, if the optimal *E*_max_ was not the same between the field sizes 15.4 × 15.4 mm^2^ and 84.6 × 84.7 mm^2^, we chose the optimal *E*_max_ for the lowest field size, because small field sizes are most predominantly used in CK treatments.

#### 2.1.2 Source FWHM optimization

The source distribution represents the distribution of photons' direction from the target. It is modeled as a Gaussian function^3^ and has one user input that is the photon source FWHM (*S*). From the results obtained using the method described in Section 2.1.1, an optimal *E*_max_ was found and used to calculate the profiles. As recommended by Accuray, we calculated the profiles for two field sizes (15.4 × 15.4 mm^2^ and 84.6 × 84.7 mm^2^) at 100 mm depth. As for the optimization of the *E*_max_, we performed the *S* optimization in two successive phases. *First phase*: the profiles were calculated for *S* of 1, 2, and 3 mm and compared to the measured ones with metrics defined hereafter. *Second phase*: from the first phase, two *S* were potentially optimal (1 and 2 mm). The profiles for *S* of 1.4, 1.6, and 1.8 mm were calculated and compared to the measured ones with the same metrics used in the first phase. The metrics used to compare the profiles were (1) the field size difference (measured at the FWHM of the profiles) obtained by the average of the FWHM of left–right and head–feet profiles, and (2) a gamma index (GI) between the measured and MC calculated profiles. The GI calculations were performed with a local 2% of maximum DD, 2 mm of maximum distance to agreement (DTA) and a dose threshold of 10% of maximum dose. These metrics were used as follows. If both the field size difference and the GI were better for one *S*, it was considered optimal. A higher GI was preferred so that if the field size difference obtained with a *S*_1_ was lower than with a *S*_2,_ but accompanied by a lower GI, then *S*_2_ was considered as optimal compared to *S*_1_. Finally, for the same reason as for the *E*_max_ optimization, the results obtained for small field size were dominant in the final decision.

### 2.2 Model validation

#### 2.2.1 Output factors

After having optimized *E*_max_ and *S*, we calculated the output factors for all field sizes with no possibility of further optimization and then compared them to the ones measured at source‐axis distance (SAD) of 800 mm. The output factors were considered acceptable if the difference was lower than 1%, as recommended by the SSRMP^7^ and AAPM^8^ in their recommendations for the quality control of medical linear accelerators.

#### 2.2.2 Phantom measurements

**FIGURE 1:**
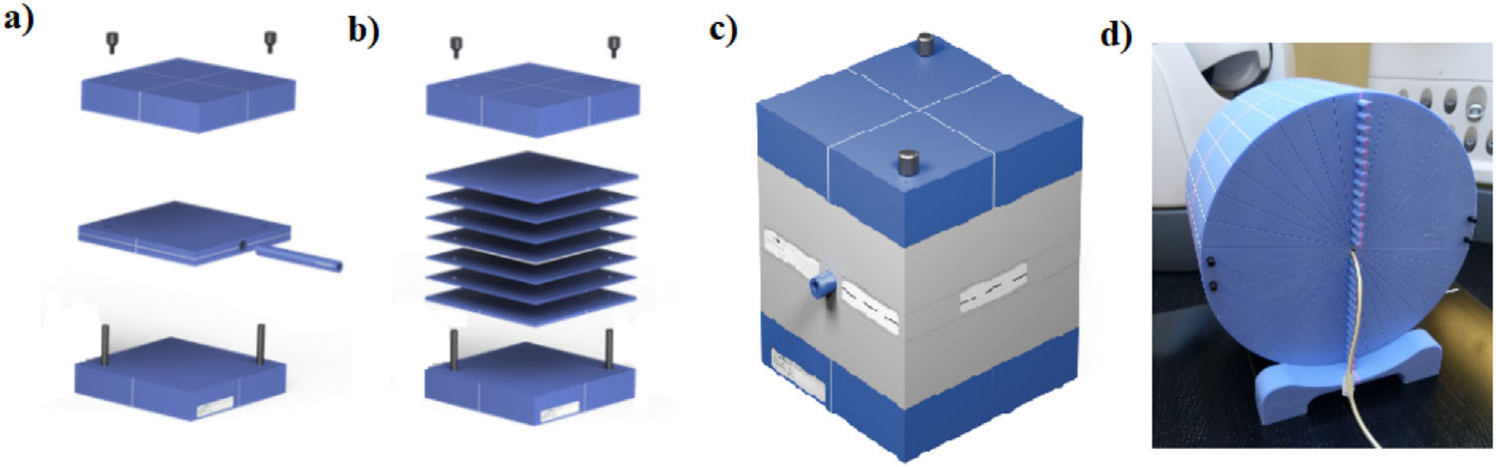
Phantoms used for validating the Monte Carlo (MC) model (a) homogeneous phantom with ionization chamber insert, (b) homogeneous phantom with films insert, (c) heterogeneous phantom with lung slabs and ionization chamber insert, and (d) homogenous phantom with different possible depths of measurements with ionization chamber inserts

We validated the MC model by comparing measured and MC calculated (with TPS) doses. The measurements were performed in four different phantoms (see Figure 1). Ionization chamber measurements (for phantoms shown in Figure 1a,c,d) were performed with an A1SL ionization chamber (Sun Nuclear, USA) metrologically traceable to the Federal Institute of metrology (METAS) and with a valid calibration for the MLC, and we used EBT3 GafChromic films (Ashland Inc., Wayne, NJ, USA) for the phantom shown in Figure 1b. For each measurement, the measured dose was corrected by the daily output variation of the CK. To calculate the dose distribution in these phantoms with the TPS, Accuray advised using the relative electron density (RED) of the phantom to set the mass density, because although the RED of the soft tissues in a human body are within 1% of their mass densities, plastic phantoms are not.^9^ The model validation consisted of six steps. From steps 1 to 5, the dose differences (Δ*D*) between the MC model calculation and the measurement were evaluated for different configurations in order to estimate the accuracy of the model. We fixed a maximum Δ*D* of ±2% to consider the model as clinically acceptable. However, a maximum Δ*D* of ±1% was expected to consider the MC model as really accurate. Step 6 consisted of calculating real treatment plans. The following sections describe each step.

##### Step 1: Single beam in homogeneous phantom (ionization chamber measurements)

The goal of this step was to compare the MC calculated and measured doses in a homogeneous phantom (Figure 1a), at the center of the beam and at 5 cm depth. For that purpose, we calculated nine plans with different equivalent square field sizes (different field sizes and/or shapes) ranging from 20.0 to 53.2 mm with a single beam (beam entrance normal to the phantom surface) in the TPS. These plans were then exported to the CK and delivered to the phantom. The dose was measured with a A1SL ionization chamber and compared to the TPS dose to obtain the dose difference Δ*D*. The closer the Δ*D* was to 0, the more accurate the MC algorithm. Additionally, to determine if the MC algorithm should be used for all clinical situations, even for homogeneous regions where the FSPB algorithm is available, we also measured the Δ*D* for four plans (among the nine previous plans) calculated with the FSPB algorithm and compared the dose differences with the ones obtained with the MC algorithm.

##### Step 2: Single beam in homogeneous phantom (film measurements)

EBT3 GafChromic films were used to verify the accuracy of the model in high‐dose gradient regions. The films were calibrated with an Elekta Synergy linear accelerator (Elekta AB, Stockholm, Sweden) against a farmer ionization chamber (Nuclear Enterprise, USA) with a 6 MV beam energy. The energy independence of the Gafchromic films^10^ made possible their use in the CyberKnife beam. The uncertainty related to film dosimetry is estimated to be ±2%.^10^ We used five plans already calculated in step 1 (with equivalent square field sizes of 14.2, 20.0, 10.1, 30.8, and 17.6 mm) to obtain fields with dimensions (in one direction) of, respectively, 10.9, 16.5, 21.2, 30.9, and 45.0 mm. The dose profiles were measured with EBT3 Gafchromic films in the homogeneous phantom (Figure 1(b)) at 5.1 cm depth. A GI between the films and the MC plans was performed for each film. The calculations were performed with a local 2% of maximum DD, 2 mm of maximum DTA, and a dose threshold of 10% of the maximum dose.

##### Step 3: Single beam in heterogeneous phantom

This step followed the same principle as step 1, but we performed the calculations and the measurements using a heterogeneous phantom with a lung insert (Figure 1c). Nine plans were created with different equivalent square field sizes ranging from 20.4 to 45.2 mm with a single beam. The dose was measured at the center of the beam at 10 cm depth with A1SL ionization chamber.

##### Step 4: Multiple beams in homogenous and heterogeneous phantoms

Four plans were calculated in the homogeneous phantom (Figure 1a) with 6, 10, 12, and 21 beams with several different entry angles. The dose measurements were performed at the center of the beam and at 5 cm in the homogeneous phantom. The same procedure was applied using a heterogeneous phantom with 6, 10, 14, and 20 beams with several different entry angles. The dose measurements were performed at the center of the beam at 10 cm depth.

##### Step 5: Different depths in homogeneous phantom

A plan with multiple beams of field sizes larger than 28.5 mm equivalent field size was calculated in the phantom showed in Figure 1d and the Δ*D* was evaluated at 3.0, 6.0, 9.0, 12.0, 15.5, and 19.5 cm depth. The phantom used for those measurements was not specific to the CK. Therefore, we inserted a fiducial on the phantom to help the positioning, but a higher uncertainty was however observed due to the impossibility to correct for rotations. To mitigate this, we performed each measurement four times, with phantom repositioning between each measurement, and compared the mean difference to the MC calculated dose.

##### Step 6: Patient‐specific QAs

Step 6 consisted on the creation of five MLC plans using the MC algorithm and the irradiation of patient‐specific QAs following our routine procedure to accept a treatment plan. The patient‐specific QA of each MLC plan was performed with the Octavius detector 1000 SRS (PTW, Germany) and a global GI of 3% of DD and 1 mm of DTA was applied. We assumed these QAs too be clinically acceptable if at least 95% of the points fulfilled the GI criteria.

## 3 RESULTS

### 3.1 Beam modeling for Monte Carlo algorithm

#### 3.1.1 Determination of the optimized *E*_max_

Table 1 presents the results obtained for the two metrics used to determine the optimal *E*_max_. According to these results, the optimal *E*_max_ was 6.4 MeV.

**TABLE 1:** Mean difference (%) between measured and Monte Carlo (MC) calculated tissue phantom ratios (TPRs), and ΔTPR% (%) for different *E*_max_ for different sets of field sizes and depths

		*E*_max_ (MeV)	6.3	6.4	6.5	6.6	6.7	6.8
First phase	15.4 × 15.4 mm^2^ and depths of 10, 15, 100, 200 and 300 mm	Mean difference (%)	−0.3	−0.2	0.4	0.9	1.0	1.1
		ΔTPR% (%)	40	80	80	40	40	40
	84.6 × 84.7 mm^2^ and depths of 10, 15, 100, 200, and 300 mm	Mean difference (%)	0.1	−0.1	0.4	0.8	0.6	1.3
		ΔTPR% (%)	80	100	100	40	60	40
Second phase	All field sizes and depths	Mean difference (%)	–*	0.5	0.6	–	–	–
		ΔTPR% (%)	–	82	72	–	–	–
	Field sizes from 7.6 × 7.7 mm^2^ to 38.4 × 38.5 mm^2^ and depths of 10, 15, 20, 50, 100, 200 mm	Mean difference (%)	–	0.2	0.3	–	–	–
		ΔTPR% (%)	–	93	87	–	–	–

* Measurements for the second phase were only performed with 6.4 and 6.5 MeV.

#### 3.1.2 Determination of the optimized source FWHM

**FIGURE 2:**
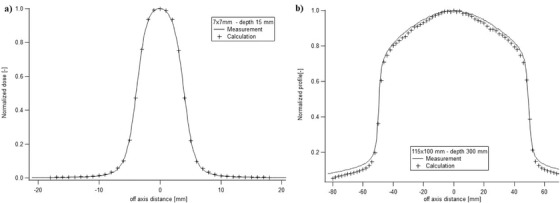
Monte Carlo (MC) calculated profiles (dashed lines) and measured profiles (filled lines) for (a) 7.6 × 7.7 mm^2^ field size at 15 mm depth and (b) 115 × 100.1 mm^2^ field size at 300 mm depth

Table 2 summarizes the different metrics used to compare the measured and MC calculated profiles. From those results, the optimized *S* was 1.8 mm. Large local DDs between the measured and the MC calculated profiles could be observed in the tail (overestimation of measured doses) and shoulder regions (underestimation of MC model), especially for large field sizes and depths. These differences became less significant for smaller field sizes and shallower depths, as seen in Figure 2.

**TABLE 2:** Field size difference and 2%/2 mm local gamma index (GI) comparison for 15.4 × 15.4 mm^2^ and 84.6 × 84.7 mm^2^ field sizes for *S* values of 1, 2, and 3 mm (first phase) and for *S* of 1.4, 1.6, and 1.8 mm (second phase)

		**15.4 × 15.4 mm^2^ field size**		**84.6 × 84.7 mm^2^ field size**	
	***S* (mm)**	**Field size difference (%)**	**GI (%)**	**Field size difference (%)**	**GI (%)**
First phase	1	−0.1	76	−0.5	98
	2	0.2	76	−0.5	98
	3	0.2	71	−0.5	100
Second phase	1.4	0.0	76	−0.4	96
	1.6	0.2	81	−0.5	98
	1.8	0.0	81	−0.5	98

### 3.2 Model validation

#### 3.2.1 Output factors

Output factors were calculated with *E*_max_ = 6.4 MeV and *S* = 1.8 mm and showed an agreement within ±0.5% when compared to the measured ones.

#### 3.2.2 Phantom measurements

##### Step 1: Single beam in homogeneous phantom (ionization chamber measurements)

The Δ*D* obtained fell between −2.2% and −1.3%. The average Δ*D* was −1.7% with a standard deviation of 0.3%. For plans calculated with FSPB algorithm, the Δ*D* fell between −1.3% and 0.4%, with an average of −0.8%.

##### Step 2: Single beam in homogeneous phantom (film measurements)

**FIGURE 3:**
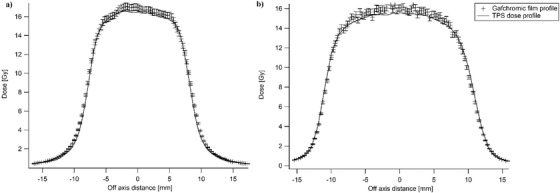
Profiles measured with EBT3 Gafchromic films (markers with error bars representing ±2% uncertainty) and calculated with the Monte Carlo (MC) model (filled line) for field sizes of (a) 16.5 mm and (b) 21.2 mm

Figure 3 presents the profiles obtained with films (markers) compared to the ones calculated with the TPS (filled lines) for 16.5 mm and 21.2 mm field sizes. Profiles for field sizes of 10.9, 30.9, and 45.0 mm can be found in Figure S1. For all field sizes, the calculated dose in the plateau region (or on‐axis for smallest field sizes) were within the ±2% of the film uncertainty, with a systematic under‐estimation (except for the 10.9 mm field size) in the MC profiles. For all profiles, the correspondence on the tails and the gradients were correct. For the field sizes of 10.9 mm and 16.5 mm, the MC profiles were slightly narrower than the measured ones; this was the contrary for the 21.2 mm field size.

##### Step 3: Single beam in heterogeneous phantom

The Δ*D* obtained for measurements with single beam in heterogeneous phantom fell between −1.7% and −0.8%, with an average of −1.2% and a standard deviation of 0.3%.

##### Step 4: Multiple beams in homogenous and heterogeneous phantoms

In the homogeneous phantom, the Δ*D* was between −1.8% and −1.2% with an average of −1.6% and a standard deviation of 0.3%. In the heterogeneous phantom, the average Δ*D* was −0.7% with a standard deviation of 0.5%. The differences were comprised between −1.3% and −0.1%. Three out of four measurements had a ΔD less than 1%.

##### Step 5: Different depths in homogeneous phantom

Table 3 shows the Δ*D* obtained at different depths of measurement. The average Δ*D* was −2.3% with a maximum difference of −3.0% at 15.5 cm depth.

**TABLE 3:** Δ*D* between measurement and TPS for different depths in homogeneous phantoms

		**Δ*D* (%)**
Depths (cm)	3.0	−1.7 ± 0.6
	6.0	−1.8 ± 0.5
	9.0	−1.7 ± 0.3
	12.0	−1.8 ± 0.2
	15.5	−3.0 ± 0.4
	19.5	−2.3 ± 0.3

##### Step 6: Re‐calculation of patient treatment plans of lung tumors

All the patient‐specific QAs performed passed our clinical criteria of acceptability with GI comprised between 95.5% and 100% with a global 1 mm/3% criteria. Four out of five QAs had GI above 98%.

## 4 DISCUSSION

### 4.1 Beam modeling for Monte Carlo algorithm

The two metrics chosen for *E*_max_ optimization and our two‐step process enabled us to unambiguously define the optimal *E*_max_. The other energies were discarded due to their higher ΔTPR% or mean difference. However, even with the optimal *E*_max_, only 82% of the points (with all field sizes and depths) of the TPRs had a difference less than ±1%, which suggested a lack of accuracy of the MC model. Optimizing the source FWHM was difficult due to two major problems. The first was caused by a lower dose than expected at the profiles’ shoulders (Figure 2b), which may have been the result of a too simple source model and the absence of a particles’ scatter model.^3^ It is a serious limitation of the MC model. Second, there were large discrepancies in the tails of the profiles due to the use of a diode for the measurements, which led to an overestimation of the measured dose.^11^ In an addendum of 2017 in the Physics Essential Guide, Accuray introduced the use of synthetic microdiamond for profile measurements in order to reduce the overestimation observed with the diode. They advised either to be aware of profile overestimation in the tails of larger beams when diodes are used or to use a synthetic microdiamond.^3^ In our case, since a diode was used to measure profiles, the correct correlation at the tail regions was verified using EBT3 Gafchromic films when we validated the model (see Section 4.2). These differences, both in the tails and shoulders, were less significant when the field size and depth were reduced, as can be seen by comparing Figure 2a,b. Another limitation of the profile optimization was a result of the user‐limited TPS module for the MC model, which prevented optimizing the profiles with both *S* and *E*_max_, although *E*_max_ had an impact on profiles.^6^ Despite these limitations, the source FWHM obtained allowed us to achieve a good compromise on gradients for different field sizes (see Section 4.2).

### 4.2 Model validation

For all field sizes, the output factors were within usual tolerances for a beam model validation.

Measurements made with a single beam in a homogeneous phantom led to a clinically acceptable Δ*D* (<±2%), although it was higher than expected for a MC algorithm in such a simple configuration. Despite the correct output factors obtained with the MC model, we did obtain a systematic dose underestimation compared to the measurements. This result highlighted the MC model's lack of accuracy. This behavior has already been reported in a previous study, where a similar dose underestimation of 1%– 2% was observed between the MC model and the measurements.^12^ The Δ*D* obtained with the MC model was not affected by the equivalent square field sizes used. Therefore, we do not recommend any restrictions on the field sizes used for MLC plans. For plans calculated with the FSPB algorithm, the average Δ*D* was lower compared to those obtained with the MC algorithm. Therefore, it seems that the FSPB algorithm is more accurate in homogeneous situations compared to the MC algorithm. Clinically, this means that for treatments in homogeneous regions, the FSPB algorithm is preferred. During step 2, profiles were acquired with EBT3 Gafchromic films. For all field sizes, the correspondence on the tails agreed between the profiles obtained from Precision with the MC model and the measured ones. This result confirmed that the large discrepancies observed during MC modeling between measured and MC calculated profiles were due to the use of a diode for the measurements. By comparing the measured and MC calculated profiles, it seems that we found a good compromise for *S* between too narrow or too large MC profiles. The film dosimetry confirmed the underestimation of the MC model seen with ionization chamber measurements. There was an exception for the smaller field size of 10.9 mm, where the dose obtained was similar to the MC dose. The average Δ*D* obtained in the heterogeneous phantom with a single beam (step 3) was slightly better than that obtained with the homogeneous phantom. Therefore, it seems that the MC algorithm has a slightly better accuracy when used in heterogeneous conditions, which is quite surprising. As for the homogeneous phantom, the Δ*D* obtained was not dependent on the equivalent square field sizes used. In step 4, multiple beam plans were created on homogeneous and heterogeneous phantoms. In the homogeneous phantom, the average Δ*D* obtained with multiple beams was the same as for the single beam. Therefore, in a homogeneous situation, the number of beams used to create a plan does not influence the accuracy of the MC model. However, in the heterogeneous phantom, we obtained a lower Δ*D* with multiple beams. This behavior has already been seen in the previous work for the fixed collimator.^5^ Therefore, the use of multiple beams in heterogeneous conditions, which is the typical situation encountered in lung treatments, led to the higher accuracy of the MC algorithm. Our step 5 investigated the accuracy of the MC model regarding the depth of measurement. For depths up to 12.0 cm, the Δ*D* obtained were similar to steps 1 and 4. However, Δ*D* increased for deeper depths. Therefore, for the treatment of tumors located at a position deeper than 12 cm, using the MC algorithm with MLC has to be used with caution. In step 6, five patient‐specific QAs of MLC plans calculated with the MC algorithm were performed. All MLC plans were clinically acceptable in term of the gamma passing rate for patient‐specific QA. Overall, the MC model could be created despite limitations related to the use of a diode, to a user‐limited interface and to an overly simplistic model. These limitations probably led to a MC model that was less accurate than could be expected, but still clinically acceptable. Our results demonstrated a better accuracy in heterogeneous conditions with multiple beams, which is clinically the most representative situation for lung treatments. Therefore, we recommend the use of the MC model only in heterogeneous conditions and we advise users to be careful regarding the depth of the target. As mentioned before, although the MC model lacks accuracy, all patient‐specific QAs largely fulfilled the criteria of acceptability, and therefore, the model is considered as clinically usable.

## 5 CONCLUSION

We created a beam model for the MC algorithm used with the MLC in the Precision CyberKnife TPS. Our study results demonstrated important differences between the MC calculations and the measurements of TPRs and profiles. However, the different configurations we tested led to dose differneces acceptable enough to consider the MC algorithm as clinically adequate, despite a lack of accuracy. All patient‐specific QAs performed fulfilled the criteria applied for clinical acceptation of treatment plans. Therefore, we conclude that using the MC model with the MLC has a benefit but should be used carefully due to a lack of accuracy.

## CONFLICT OF INTEREST

The author declares that there is no conflict of interest that could be perceived as prejudicing the impartiality of the research reported.

## AUTHOR CONTRIBUTIONS

All authors have made substantial contributions to conception and design, or acquisition of data, or analysis and interpretation of data, have been involved in drafting the manuscript or revising it critically for important intellectual content, have given final approval of the version to be published, have participated sufficiently in the work to take public responsibility for appropriate portions of the content, and have agreed to be accountable for all aspects of the work in ensuring that questions related to the accuracy or integrity of any part of the work are appropriately investigated and resolved.

## Supporting information


**FIGURE S1** Profiles measured with EBT3 Gafchromic films (dashed lines with error bars representing ±2% uncertainty) and calculated with the MC model (filled line) for field sizes of (a) 10.9 mm, (b) 30.9 mm, and (c) 45.0 mm
