# Supplementary material for: Validation of Monte Carlo dose calculation algorithm for CyberKnife multileaf collimator
Source: J Appl Clin Med Phys. 2021 Dec 1;23(2):e13481. doi: 10.1002/acm2.13481 (PMC8833269; doi:10.1002/acm2.13481)
Supplement: Supplementary file 1 — FIGURE S1 Profiles measured with EBT3 Gafchromic films (dashed lines with error bars representing ±2% uncertainty) and calculated with the MC model (filled line) for field sizes of (a) 10.9 mm, (b) 30.9 mm, and (c) 45.0 mm [file ACM2-23-e13481-s001.docx]

**SUPPLEMENTARY FIGURES AND FIGURE LEGENDS**


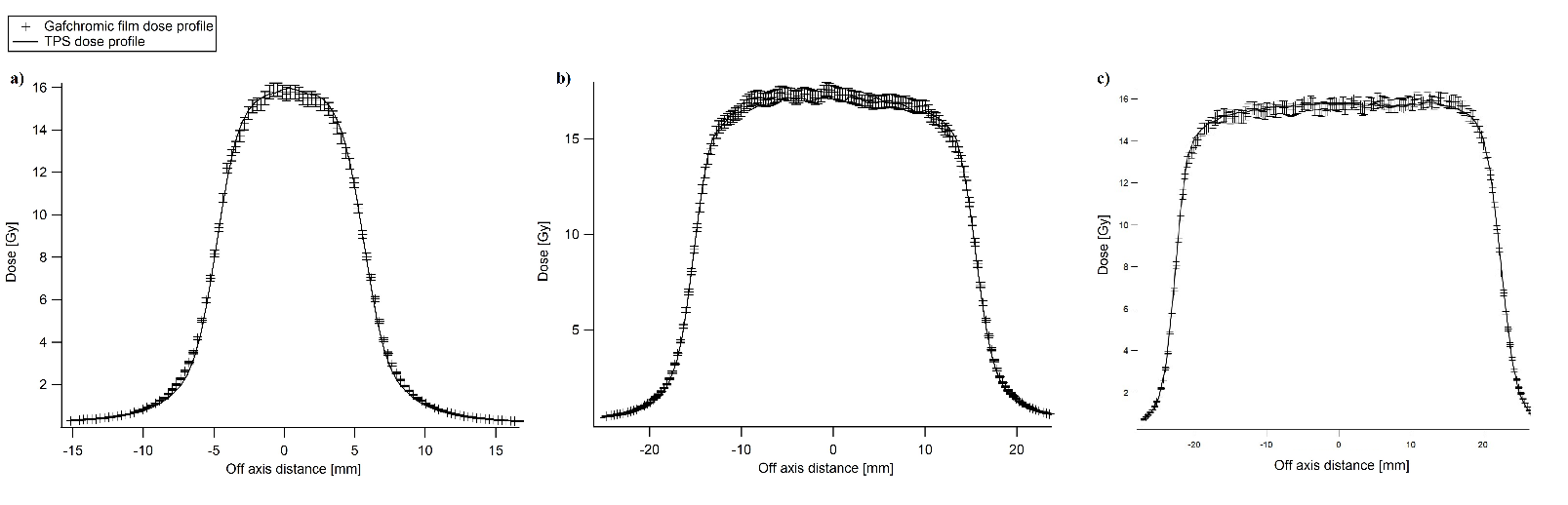


Supplementary Fig 1. Profiles measured with EBT3 Gafchromic films (dashed lines with error bars representing ±2% uncertainty) and calculated with the MC model (filled line) for field sizes of a) 10.9mm, b) 30.9mm and c) 45.0mm.
